# Financial Stress and Risk for Entry into Medicaid Among Older Adults

**DOI:** 10.1093/geroni/igz040

**Published:** 2019-10-16

**Authors:** Amber Willink, Jennifer L Wolff, John Mulcahy, Karen Davis, Judith D Kasper

**Affiliations:** Department of Health Policy and Management, Johns Hopkins Bloomberg School of Public Health, Baltimore, Maryland

**Keywords:** Long-term services and supports, Public programs, Social determinants of health

## Abstract

**Background and Objectives:**

Spending in the Medicaid program is a significant concern to both state and federal policy makers. Medicaid spending is driven by program enrollment and services use. Older adults with high health care needs incur a disproportionate proportion of program spending. This analysis identifies factors that place older Medicare beneficiaries at increased risk for entering into Medicaid.

**Research Design and Methods:**

We use multinomial logistic regression and the 2011–2017 National Health and Aging Trends Study (NHATS) to examine the risks among older Medicare beneficiaries for entering into Medicaid over a 6-year follow-up period. We examine both time-invariant and time-varying factors to measure the impact of social and health and functioning changes at older ages.

**Results:**

The risk of entry into Medicaid was higher for older adults who relocated to a nursing home (relative risk ratio [RRR]: 7.75; 95% confidence interval [CI]: 5.33–11.26) or other residential care setting (RRR: 1.36; 95% CI: 0.96–1.92) compared to those who remained in traditional community settings. Older adults who reported skipping a meal in the last month because there was not enough money to buy food were 2.4 times (95% CI: 1.10–5.21) more likely to enter Medicaid than those who did not. Similarly, older adults who reported not having enough money to pay household utility bills in the last year were 1.89 times (95% CI: 1.08–3.30) more likely to enter Medicaid.

**Discussion and Implications:**

Study findings suggest that trouble paying for basic needs increases the risk of entry into Medicaid. Further research is required to examine whether addressing these needs through improved access to social services that enable older adults to live safely in their home may delay or mitigate entry into Medicaid.

**Translational Significance:**

This analysis suggests that experiencing financial stress for the basic needs, such as food, or utilities, increases the risk for entry into Medicaid which has greater spending implications for state budgets. Some states are engaging in efforts to address the social determinants of health, such as food and housing availability, among low-income populations not yet eligible for Medicaid to avoid or delay entry into Medicaid.

Medicaid, the health insurance safety net program in the United States, covers one in five Americans and accounts for 17% of national health expenditures (Cubanski et al., 2015; Cuckler et al., 2018). Spending growth in the Medicaid program is expected to be a substantial contributor to national health spending increases in the next 10 years, primarily due to a greater proportion of older adults with high health care and long-term services and supports (LTSS) needs enrolling in the program (Bennett, Curtis, & Harrod, 2018; Cuckler et al., 2018). Medicaid is a state-run program financed by both federal and state governments. The consequences of more individuals enrolling into Medicaid are therefore increased costs to both state and federal governments. While there is significant interest on the part of policy makers to curb the growth in health care spending in the Medicaid program, the emphasis has primarily been on cost containment strategies of those within the program such as block grants or cost containment mechanisms such as waivers and waiting lists, rather than mitigating the risk of entering Medicaid. This analysis seeks to identify factors that place older Medicare beneficiaries at risk for Medicaid.

Previous studies have identified many factors that contribute to the risk of Medicaid entry among older adults. Long-term nursing home use is a consistent predictor of Medicaid entry among older adults as very few Americans plan ahead for their long-term care needs or are aware of how costly these services can be (Tell, 2011; Wiener, Anderson, Khatutsky, Kaganova, Keeffe, et al., 2013). For example, a semiprivate room in a nursing home costs approximately $90,000 per year (Genworth, 2017), whereas the median income and savings of older adults are $26,000 and $74,000, respectively (Jacobson, Griffin, Neuman, & Smith, 2017).

While LTSS needs have long been a driver for Medicaid entry among older adults, now only half of those who enter Medicaid are receiving some LTSS (Wiener, Anderson, Khatutsky, Kaganova, & O’Keeffe, 2013). Recent studies have sought to better understand what other drivers there may be for entry into Medicaid, with many finding high or unexpected health care costs, particularly those paid for out-of-pocket as increasing the risk for Medicaid entry (Keohane, Trivedi, & Mor, 2017, 2018; Willink, Davis, Schoen, & Wolff, 2016). They also acknowledge, however, that approximately half of those who entered Medicaid did not have any health spending. Other costs that have yet to be quantified for their impact on risk of entry into Medicaid are those stemming from social determinants of health such as food- and housing-related expenses.

This study contributes to existing understanding of risk of Medicaid entry among older adults in three important ways. Firstly, we are able to assess annual changes in measures of financial stress driven by social determinants of health, such as housing and food accessibility, may affect risk of Medicaid entry. Previous studies of risk of Medicaid entry have focused primarily on health needs (Willink, Davis, & Schoen, 2016) or income and asset spend down (Wiener, Anderson, Khatutsky, Kaganova, Keeffe, et al., 2013; Wiener, Anderson, Khatutsky, Kaganova, & O’Keeffe, 2013). This work contributes to the understand of risk for Medicaid entry by broadening the scope of risk factors to include financial stress driven by social determinants of health. Secondly, this study provides novel contributions to the existing literature through the use of timely data collected through the National Health and Aging Trends Study (NHATS). Given the changing policy and service delivery landscape, contemporary information to examine this issue is critical. The NHATS collects annual longitudinal data on a nationally representative sample of the Medicare population aged 65 and older, thereby enabling study of risks over time. Using the NHATS, this study provides a better characterized sample by functional status, cognitive impairment, living arrangements, and LTSS use on an annual basis. Finally, the multinomial logistic regression analysis with time-varying covariates controls for and estimates the effect of changes in personal health and circumstances over the follow-up period, and accounts for competing risks that older adults experience over the 6-year period. These three novel features all contribute to improving our current understanding of risk of entry into Medicaid and provide new insight into potential mechanisms to decrease risk of Medicaid entry or possible target populations at high risk of entry.

## Design and Methods

### Study Sample

**Figure 1.**
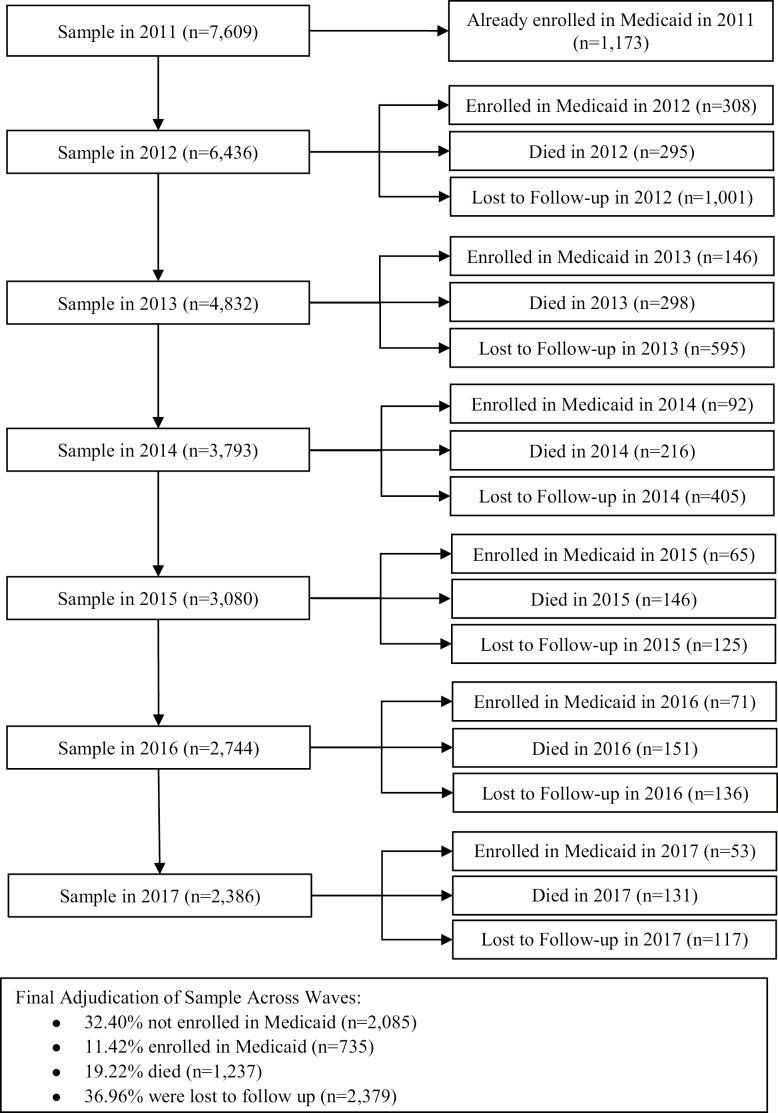
Construction of longitudinal analytic sample of older adults 2011–2017.

This study uses 2011–2017 NHATS to examine the risks longitudinally among older Medicare beneficiaries for entering into Medicaid over a 6-year follow-up period. The NHATS is a nationally representative study of Medicare beneficiaries aged 65 and older that provides in-depth information on functional and cognitive status and how older adults are accommodating impairments and functional declines in their daily lives. The NHATS applies a multistage sampling design using the Medicare enrollment file as a sampling frame (Kasper & Freedman, 2017). It oversamples Medicare beneficiaries at older ages, as well as black individuals. A total of 7,609 Medicare beneficiaries living in the community or residential care were interviewed in 2011, and subsequently reinterviewed annually between 2011 and 2017. While individuals living in a nursing home in 2011 only received a facility interview rather than the full questionnaire, if participants transitioned to a nursing home over the follow-up period (2011–2017) they were eligible for interview. This analysis excludes participants who reported being covered by Medicaid at the time of the baseline interview in 2011 (*n* = 1,173). Eligible participants are subsequently categorized as enrolled or not enrolled in Medicaid at each wave or are censored from the study in the wave they experience loss to follow-up or death. Figure 1 illustrates the study sample followed between 2011 and 2017, their time at risk of experiencing the event (Medicaid entry), and censoring events (death and lost to follow-up).

### Variables/Measures

Both time-invariant and time-variant variables are included in the analysis of Medicaid entry. Time-invariant independent variables measured at baseline in 2011 include age, gender, race and ethnicity, education (less than high school [HS], HS graduate, more than HS), income relative to the federal poverty level (FPL) as a binary measure (<200% FPL or ≥200% FPL), and home ownership (rent or own home).

Time-varying factors include marital status (married/partnered vs not married/partnered), place of residency (private household, non-nursing home residential care community, nursing home), number of activities of daily living (ADLs) and instrumental activities of daily living (IADLs) for which receiving help, dementia status, using assistive devices (yes/no), numbers of chronic conditions, and financial stress variables. ADLs include receiving help with eating, bathing, dressing, toileting, transferring in and out bed or a chair, and inside mobility. IADLs include help with meal preparation, grocery shopping, transportation, banking, medication management, and laundry, for health and functioning reasons. The indicator of probable dementia (yes/no) is based on a composite of self-report of a doctor’s diagnosis of dementia or Alzheimer’s disease, cognitive testing, and responses to a proxy informant screening instrument (Kasper, Freedman, & Spillman, 2013). Measures of financial stress include whether in the last month someone skipped any meals because there was not enough food, or money to buy food. Participants are also asked whether there were times in the last year when they did not have enough money to pay the rent or mortgage, pay utilities bills, or pay medical or prescription drug bills. For the longitudinal analysis of risk to entry into Medicaid in any given year, we examine the contribution of each financial stress variable separately.

The outcome variable of interest is entry into Medicaid. In each wave of the survey, participants are asked questions about insurance coverage. In this study, entry into Medicaid is determined by a participant’s self-report of being covered by Medicaid at the time of the interview.

### Analytic Methods

First, we describe baseline characteristics of study participants who did and did not enter Medicaid over the follow-up period. Differences between groups were measured by baseline characteristics using the chi-squared test for categorical variables and *t* test for comparing means of continuous variables.

Second, we employed multinomial logistic regression to analyze the risk of entry into Medicaid. Multinomial logistic regression was chosen due to the high proportion of censoring events across the 6 years due to loss to follow-up and death, which act as competing risks to the outcome of interest, Medicaid entry. The data for this analysis are organized in person-time form, that is, one observation per person per year at risk of Medicaid entry, to account for changes in the time-varying covariates over the follow-up period. Our sample of 6,436 participants contributed 20,579 person-years across the 6-year observation period from 2011 through 2017. Covariate and outcome (Medicaid entry) values are assessed in the same measurement year due to the reliance on survey-related information. The analysis therefore examines associations between covariates and Medicaid entry and does not assess causality. All analyses include sampling weights from the 2011 NHATS to account for the sampling strategy employed in the survey. The regression models also account for repeated measures across individuals in time.

## Results

Every year of the 6-year follow-up, between 2.43% and 4.79% of the surveyed population entered Medicaid with a total of 11.42% of the original sample entering Medicaid over the entire duration of the observation period (Figure 1). Table 1 presents a summary of study sample characteristics at baseline and compares the proportion of those who did and did not enter Medicaid over the follow-up period. Those who entered into Medicaid over the 6-year follow-up period from 2011 to 2017 were more likely to be black (16.43% vs 5.44%), or Hispanic (11.35% vs 4.31%) compared to those who did not enter Medicaid. Older adults with less than HS education were more likely to enter Medicaid than those with HS education or more (38.46% vs 14.86%). Older adults with lower incomes (income relative to the FPL below 200%) were also more likely to enter Medicaid than higher-income older adults (63.14% vs 24.87%). Fewer persons who entered into Medicaid over the follow-up period were married or partnered at baseline (45.10% vs 62.09%) compared to those who did not enter Medicaid.

**Table 1.** Baseline Characteristics of Older Adults Who Do and Do Not Enter into Medicaid over Six Years (2011–2017)

Baseline characteristics	Do not enter into Medicaid	Enter into Medicaid	*p* Value
Age			
65–69	29.09% (27.95%, 30.25%)	24.29% (20.44%, 28.62%)	
70–74	25.00% (23.99%, 26.04%)	20.28% (16.89%, 24.16%)	
75–79	18.70% (17.76%, 19.67%)	23.43% (20.11%, 27.11%)	
80–84	14.53% (13.73%, 15.37%)	15.96% (13.25%, 19.1%)	
85–89	8.65% (7.98%, 9.35%)	10.79% (8.84%, 13.11%)	
90+	4.04% (3.58%, 4.55%)	5.24% (3.86,7.07%)	<.01
Female	55.33% (53.60%, 57.04%)	60.01% (56.21%, 63.69%)	.03
Race/ethnicity			
White, non-Hispanic	86.53% (85.15%, 87.81%)	67.09% (62.23%, 71.62%)	
Black, non-Hispanic	5.44% (4.75%, 6.22%)	16.43% (13.89%, 19.32%)	
Hispanic	4.31% (3.59%, 5.17%)	11.35% (8.19%, 15.52%)	
Other, non-Hispanic	3.72% (2.99%, 4.62%)	5.13% (3.11%, 8.34%)	<.001
Education			
Less than HS	14.86% (13.40%, 16.46%)	38.46% (33.86%, 43.28%)	
HS graduate	27.47% (25.88%, 29.13%)	29.51% (25.59%, 33.75%)	
More than HS	56.38% (53.94%, 58.79%)	30.05% (25.49%, 35.04%)	<.001
Income less than 200% FPL^a^	24.87% (23.23%, 26.58%)	63.14% (58.45%, 67.59%)	<.001
Owns home	79.83% (78.48%, 81.11%)	58.18% (52.99%, 63.20%)	<.001
Residential community	4.63% (3.88%, 5.51%)	7.81% (5.32%, 11.32%)	<.01
Married/partnered	62.09% (60.56%, 63.60%)	45.10% (40.97%, 49.31%)	<.001
Probable dementia^b^	7.73% (6.98%, 8.54%)	16.04% (13.26%, 19.26%)	<.001
Uses assistive devices	55.37% (54.10%, 56.64%)	63.05% (58.43%, 67.44%)	<.01
Trouble paying rent^c^	1.26% (0.91%–1.73%)	4.23% (2.66%–6.69%)	<.001
Trouble paying utilities^c^	1.59% (1.23%–2.06%)	6.98% (4.97%–9.72%)	<.001
Trouble paying for food^c^	0.33% (0.19%–0.56%)	2.38% (1.24%–4.54%)	<.001
Trouble paying for medical care^c^	1.88% (1.48%–2.39%)	7.91% (5.62%–11.02%	<.001
Mean number of chronic conditions^d^ (std err)	2.41 (0.02)	2.68 (0.07)	<.01
Mean number of ADLs^e^ (std err)	0.29 (0.01)	0.46 (0.05)	<.001
Mean number of IADLs^f^ (std err)	0.41 (0.01)	0.73 (0.05)	<.001

Source: National Health and Aging Trends Study, 2011–2017.

*Note*. ADL = activities of daily living; FPL = federal poverty level; HS = high school; IADL = instrumental activities of daily living; std err = standard error.

^a^In 2011 200% of the FPL was $21,576 for an individual, and $27,192 for a couple.

^b^Probable dementia is a variable developed within the NHATS to identify probable dementia based on a composite of self-report of a doctor’s diagnosis of dementia or Alzheimer’s disease, cognitive testing, and responses to AD8 Dementia Screening Interview by proxies.

^c^Financial stress variables were first collected in 2012.

^d^Chronic conditions include heart attack, heart disease, high blood pressure, arthritis, osteoporosis, diabetes, lung disease, stroke, and cancer.

^e^ADLs include receiving help with eating, bathing, dressing, toileting, transferring in and out of bed, and inside mobility.

^f^IADLs include receiving help with grocery shopping, preparing hot meals, medication management, banking, transportation, and laundry.

A greater proportion of older Medicare beneficiaries who entered into Medicaid had high health needs at baseline compared to those who did not enter Medicaid (Table 1). This includes a higher average number of chronic conditions (2.68 vs 2.41), ADLs (0.46 vs 0.29), IADLs (0.73 vs 0.41), probable dementia (16.04% vs 7.73%), and a higher proportion who reported use of assistive devices for ADLs (63.05% vs 55.37%). Across all financial stress variables, the proportion who could not pay rent, utilities, meals, and medications was larger among those who entered Medicaid during the follow-up period.

We use multinomial logistic regression analysis to examine the role of time-varying factors such as place of residence, health status, functioning, and financial stress on the risk of entry into Medicaid (Table 2) at any time point over the observation period while controlling for time-invariant factors. In the fully adjusted model, the most impactful change that bore a 7.75 times higher risk of entry into Medicaid was moving into a nursing home compared to living in a private residence (relative risk ratio [RRR]: 7.75; 95% confidence interval [CI]: 5.33–11.26). Moving to a non-nursing home residential care community was also associated with a 36% higher risk of entry into Medicaid albeit not statistically significant (RRR: 1.36; 95% CI: 0.96–1.92). Those with two or more IADLs carry a 54% increased relative risk for entering Medicaid (IADLs RRR: 1.54; 95% CI: 1.21–1.95). Other health status variables such as the number of chronic conditions, or cognitive impairment were not associated with an increased risk of entry into Medicaid. The risk of entry into Medicaid was significantly greater for those who experienced trouble paying for utilities (RRR: 1.89; 95% CI: 1.08–3.3) and trouble paying for food (RRR: 2.39; 95% CI: 1.10–5.21).

**Table 2.** Adjusted Relative Risk Ratio (RRR) for Entry into Medicaid Between 2011 and 2017

	RRR (95% CI)	*p* Value
Time-invariant factors (2011)		
Age		
65–69	Reference	
70–74	0.87 (0.65–1.16)	.33
75–79	1.10 (0.84–1.45)	.48
80–84	0.90 (0.67–1.20)	.46
85–89	0.81 (0.58–1.13)	.21
90+	0.67 (0.45–1.00)	.05
Women	0.87 (0.71–1.06)	.17
Race/ethnicity		
White, non-Hispanic	Reference	
Black, non-Hispanic	2.61 (2.14–3.17)	<.001
Hispanic	2.31 (1.66–3.22)	<.001
Other, non-Hispanic	1.91 (1.12–3.27)	.02
Education		
Less than HS	2.62 (2.05–3.34)	<.001
HS graduate	1.71 (1.35–2.16)	<.001
More than HS	Reference	
Income less than 200% FPL^a^	2.59 (2.09–3.21)	<.001
Rents home	1.68 (1.37–2.05)	<.001
Time-varying factors		
Not married or partnered	1.06 (0.85–1.32)	.61
Place of residence		
Private residence	Reference	
Residential community	1.36 (0.96–1.92)	.08
Nursing home	7.75 (5.33–11.26)	<.001
Probable dementia^b^	1.12 (0.88–1.44)	.35
Uses assistive devices	1.06 (0.84–1.32)	.64
Two or more ADLs^c^	1.51 (1.12–2.02)	.01
Two or more IADLs^d^	1.54 (1.21–1.95)	<.001
Number of chronic conditions^e^	1.01 (0.87–1.16)	.94
Any financial stress^f^		
Trouble paying rent	0.94 (0.49–1.77)	.84
Trouble paying utilities	1.89 (1.08–3.30)	.03
Trouble paying for food	2.40 (1.10–5.20)	.03
Trouble paying for medical care	1.25 (0.77–2.03)	.37

Source: National Health and Aging Trends Study, 2011–2017.

*Note.* ADL = activities of daily living; CI = confidence interval; FPL = federal poverty level; HS = high school, IADL = instrumental activities of daily living; Reference = reference group.

^a^In 2011 200% of the FPL was $21,576 for an individual, and $27,192 for a couple.

^b^Probable dementia is a variable developed within the NHATS to identify probable dementia based on a composite of self-report of a doctor’s diagnosis of dementia or Alzheimer’s disease, cognitive testing, and responses to AD8 Dementia Screening Interview by proxies.

^c^ADLs include receiving help with eating, bathing, dressing, toileting, transferring in and out of bed, and inside mobility.

^d^IADLs include receiving help with grocery shopping, preparing hot meals, medication management, banking, transportation, and laundry.

^e^Chronic conditions include heart attack, heart disease, high blood pressure, arthritis, osteoporosis, diabetes, lung disease, stroke, and cancer.

^f^Financial stress includes whether in the last month they skipped any meals because there was not enough food, or money to buy food; whether there were times in the last year when they did not have enough money to pay the rent or mortgage; pay utilities bills; or pay medical or prescription drug bills. Financial stress variables were reported in waves 2012–2017.

Of the time-invariant factors, older adults with below median incomes at baseline and those who rented, rather than owned, their homes were more likely to enter into Medicaid over the follow-up period (RRR: 2.59; 95% CI: 2.09–3.21, and RRR: 1.68; 95% CI: 1.37–2.06, respectively). African American and Hispanic Americans were more like to enter Medicaid compared to white Americans (RRR: 2.61; 95% CI: 2.14–3.17, and RRR: 2.31; 95% CI: 1.66–3.22, respectively). Education was also a protective factor against entering into Medicaid- older adults who did not graduate HS had higher odds of entering Medicaid than those who went to college (RRR: 2.62; 95% CI: 2.05–3.34). Full results of competing risk calculations are included as Supplementary Material.

## Discussion and Implications

This study finds that relocating from traditional community settings to a nursing home continues to be a dominant factor to increasing one’s risk of Medicaid entry. Unlike previous studies, we also examined the role of financial stress on Medicaid entry. Financial stress, particularly in the form of not being able to pay for household utilities, or not being able to pay for food, is also associated with greater risk of entry into Medicaid. Taken together, this study highlights the financial vulnerability of at-risk subgroups of older adults and identifies possible policy-relevant targets that may reduce entry into Medicaid.

The high odds of entry into Medicaid among those in nursing homes is consistent with other studies that have examined this issue (Wiener, Anderson, Khatutsky, Kaganova, Keeffe, et al., 2013; Wiener, Anderson, Khatutsky, Kaganova, & O’Keeffe, 2013). In many states, the only guaranteed support for older adults who need LTSS but cannot afford them in community settings is to enter a nursing home, spend down income and assets, and qualify for Medicaid. In 2007, the national Money Follows the Person (MFP) demonstration was initiated to support the transition of eligible Medicaid enrollees living in nursing homes and institutions back to living in the community. By the end of 2015, the MFP demonstration had transitioned more than 63,000 Medicaid enrollees back to the community. While this program has been paramount to efforts to rebalance LTSS service delivery from institutions to the community, it only manages to transition 1% of the population eligible for transition back to the community annually with older adults having the lowest transition rates, and it does it once individuals have already diminished their income and assets and become eligible for Medicaid (Irvin et al., 2017). Instead of relying on retroactive programs like MFP to eventually provide cost savings by transitioning persons back to the community, states are beginning to think proactively about how to avoid nursing home placement and promote community-based supports in order to avoid possible spend down to Medicaid. Some states, such as Washington (Washington State Department of Social and Health Services, 2018) and Minnesota (MN Department of Human Services, 2017), have waiver programs designed to provide support to older adults who do not yet qualify for the Medicaid program but do not have sufficient incomes to support self-care needs in the community. Minnesota reports that in 2016 the average cost of supporting an enrollee living in the community through the waiver was $886 per month, whereas the alternative of having them spend down to become eligible for Medicaid would cost $6,783 per enrollee per month in the community (MN Department of Human Services, 2017).

There has also been a policy shift to consider the social determinants of health which include, but are not limited to, issues of housing stability and food insecurity (Alley, Asomugha, Conway, & Sanghavi, 2016). The CHRONIC Care Act that passed in early 2018 as part of the Bipartisan Budget Act gives Medicare Advantage plans the flexibility to include nonmedical services as part of supplemental benefits (“Bipartisan Budget Act of 2018,” 2018; Willink & DuGoff, 2018). This could include support for a variety of services such as meal delivery, transportation services, personal care services that could support the improvement or maintenance of health or functioning, and would also alleviate some of the financial pressure or stress experienced by Medicare beneficiaries with these needs. Recent studies of participation in social programs, such as the Supplemental Nutrition Assistance Program (SNAP) and the Low-Income Home Energy Assistance Program, indicate that receiving benefits and greater generosity of benefits is protective against nursing home placement and adverse health outcomes (Berkowitz, Seligman, Rigdon, Meigs, & Basu, 2017; Berkowitz et al., 2018; Samuel et al., 2018; Szanton et al., 2017). Further research is required to examine whether greater participation in social programs like SNAP or LIHEAP, or access to nonmedical services could delay or mitigate entry into Medicaid. Cost–benefit research is needed to determine whether addressing the causes of financial stress, such as access to low-cost housing, meal programs, or reduced out-of-pocket burden for medical expenses, yields downstream savings and to whom. The challenge is that the agencies that stand to benefit from participation in these social programs are not the ones currently paying for the programs (Nichols & Taylor, 2018).

The findings of this study also highlight the greater likelihood of Medicaid entry among those with functional limitations. Given the lack of private planning for LTSS needs, or a public insurance option to support LTSS needs, Medicaid has long been the primary payer of LTSS in the United States (Chernof et al., 2013; Feder & Komisar, 2012; Tell, 2013). The significant out-of-pocket costs associated with paying for LTSS lead many older adults with functional limitations to spend down income and assets to qualify for Medicaid. In the 10-year projections of the National Health Expenditures, spending in the Medicaid program is expected to increase due to the complexity of needs among new enrollees (Cuckler et al., 2018). Many LTSS financing alternatives (Davis, Willink, & Schoen, 2016; Favreault, Gleckman, & Johnson, 2015; Bipartisan Policy Center, 2016; The Long-Term Care Financing Collaborative, 2016) have been proposed to better support older adults and relieve the pressure being placed on the Medicaid program to meet the needs of older adults with functional limitations, although these proposals have yet to gain any political interest or momentum. It is too early to tell whether the potential expanded benefits available under Medicare Advantage plans, legislated in the CHRONIC Care Act, will offer some financial protection from LTSS costs (Willink & DuGoff, 2018).

### Limitations

A limitation of this study is that it relies on self-report of Medicaid coverage. Studies have shown that prevalence of Medicaid coverage varies when using self-report compared to administrative claims data, often undercounting the number of individuals enrolled. This is exacerbated when the recall time of the question is long (Call, Davern, Klerman, & Lynch, 2013). The point-in-time reference period used in the NHATS therefore reduces this measurement error. Given there is some transition in and out of Medicaid eligibility, there is the possibility that using this point-in-time reference period also undercounts Medicaid coverage, although income fluctuations that can affect eligibility are less likely among older people. Another limitation is the discrete time nature of these data so that we are unable to discern whether changes in the time-varying covariates between interviews occurred before or after the individual became covered by Medicaid.

## Conclusion

As the population ages, more individuals will experience functional and/or cognitive impairment at some point that hinders independent living. These needs come with significant medical and nonmedical expenses which are placing great financial stress on older adults. This study suggests that other financial stresses beyond health care, such as food insecurity and housing, may be placing older adults at greater risk for entry into Medicaid. The result will be higher Medicaid outlays placing demands on federal and state government budgets. Further research is needed to examine whether addressing the social determinants of health among older adults can delay or avoid entry into Medicaid among at-risk adults.

## Supplementary Material

igz040_suppl_Appendix
